# Gallic Acid-Functionalized, TiO_2_-Based Nanomaterial—Preparation, Physicochemical and Biological Properties

**DOI:** 10.3390/ma15124177

**Published:** 2022-06-13

**Authors:** Pawel Bakun, Beata Czarczynska-Goslinska, Dariusz T. Mlynarczyk, Marika Musielak, Kinga Mylkie, Jolanta Dlugaszewska, Tomasz Koczorowski, Wiktoria M. Suchorska, Marta Ziegler-Borowska, Tomasz Goslinski, Rafal Krakowiak

**Affiliations:** 1 Chair and Department of Chemical Technology of Drugs, Poznan University of Medical Sciences, Grunwaldzka 6, 60-780 Poznan, Poland; mlynarczykd@ump.edu.pl (D.T.M.); tkoczorowski@ump.edu.pl (T.K.); rafal.krakowiak@student.ump.edu.pl (R.K.); 2 Doctoral School, Poznan University of Medical Sciences, Bukowska 70, 60-812 Poznan, Poland; marika.musielak@wco.pl; 3 Chair and Department of Pharmaceutical Technology, Poznan University of Medical Sciences, Grunwaldzka 6, 60-780 Poznan, Poland; bgoslinska@ump.edu.pl; 4 Department of Electroradiology, Poznan University of Medical Sciences, Garbary 15, 61-866 Poznan, Poland; wiktoriasuchorska@ump.edu.pl; 5 Faculty of Chemistry, Nicolaus Copernicus University in Torun, Gagarina 7, 87-100 Torun, Poland; kinga.mylkie@doktorant.umk.pl (K.M.); martaz@umk.pl (M.Z.-B.); 6 Chair and Department of Genetics and Pharmaceutical Microbiology, Poznan University of Medical Sciences, Swiecickiego 4, 60-781 Poznan, Poland; jdlugasz@ump.edu.pl

**Keywords:** DPPH assay, fibroblasts, MTT assay, photocytotoxicity, photodynamic antimicrobial chemotherapy

## Abstract

Wound healing and skin tissue regeneration remain the most critical challenges faced by medical professionals. Titanium(IV) oxide-based materials were proposed as components of pharmaceutical formulations for the treatment of difficult-to-heal wounds and unsightly scarring. A gallic acid-functionalized TiO_2_ nanomaterial (TiO_2_-GA) was obtained using the self-assembly technique and characterized using the following methods: scanning electron microscopy (SEM), transmission electron microscopy (TEM), nanoparticle tracking analysis (NTA), X-ray powder diffraction (XRPD), infrared spectroscopy (IR), Raman spectroscopy and thermogravimetry (TG). Additionally, physicochemical and biological tests (DPPH assay, Microtox^®^ acute toxicity test, MTT assay) were performed to assess antioxidant properties as well as to determine the cytotoxicity of the novel material against eukaryotic (MRC-5 pd19 fibroblasts) and prokaryotic (*Staphylococcus aureus, Escherichia coli, Candida albicans, Aliivibrio fischeri*) cells. To determine the photocytotoxicity of the material, specific tests were carried out with and without exposure to visible light lamps (425 nm). Following the results, the TiO_2_-GA material could be considered an additive to dressings and rinsing suspensions for the treatment of difficult-to-heal wounds that are at risk of bacterial infections.

## 1. Introduction

Wound healing has historically been a challenging problem in medicine. Damaged tissue is at risk of possible infection, which may lead to severe complications, including gangrene and amputations, in extreme cases [1]. Even today, mistreated wounds can be a cause of morbidity and mortality [2]. A wound can be formed either by an internal pathological process or by external influence, i.e., mechanical disruption of the skin. Wounds can range from a simple fractioning of skin to deeper tissue damage: subcutaneous tissue, muscles, tendons and ligaments, vessels, nerves and even bones [3]. Apart from acute wounds, many hard-to-heal chronic wounds are formed as a result of direct or indirect damage of the cutaneous coverage, including arterial, venous, diabetic and pressure ulcers [4]. Several factors contribute to the wound-healing process, such as multiple cell populations, growth factors or inflammation mediators, etc. [5,6]. The mechanism of physiological wound healing can be divided into four partially overlapping stages: (i) coagulation and haemostasis; (ii) inflammation; (iii) proliferation; and (iv) wound remodeling with scar tissue formation [3]. Each of these stages can be strongly supported by a specific treatment.

The conventional treatment of a wound is based on aseptic wound care (debridement) and standard cotton dressing materials such as gauze and bandages. However, nowadays, wound healing treatment is being developed by the introduction of advanced materials based on, e.g., silver nanoparticles [7] and new therapeutic approaches such as hyperbaric therapy [8], which are often used to support wound healing. Many advanced materials designed to tackle the problem of wound healing have recently been studied, including various biopolymers such as alginate, chitosan, hyaluronan, pectin and gelatin [9]. Moreover, the performance of titanium(IV) oxide nanoparticles incorporated in gellan gum in wound healing has been assessed [10].

Titanium(IV) oxide (titania, TiO_2_) is a white inorganic compound that occurs in nature in three crystal forms: (a) anatase, (b) rutile and (c) brookite. The first two polymorphic forms of TiO_2_ can be easily prepared and reveal good photoactivity, and thus broad potential applicability [11]. The main features determining the wide interest in these materials are high photostability and photocatalytic activity, low toxicity and cost [12]. However, the optical response of TiO_2_ in the UV light range is accompanied by fast recombination of the generated electron-hole pairs, thus constituting a significant limitation, which can be overcome by surface modification of titania [13,14]. The photocatalytic activity of titania in the visible light range can be achieved by doping with metal oxides or by surface modifications with various sensitizers, including graphene, carbon nanotubes and porphyrinoids [13,15,16,17]. Titania revealed many potential applications in pharmacy and medicine due to its photocatalytic properties and oxidizing activity [18,19]. For example, it was evaluated in new treatments of skin diseases, such as acne and atopic dermatitis [20], and has also been used as a antimicrobial agent. For example, Gupta et al. assessed TiO_2_ and Ag-TiO_2_ in terms of their antibacterial properties against *Escherichia coli*, *Pseudomonas aeruginosa* and *Staphylococcus aureus* [21]. Moreover, the antibacterial activity against *E. coli* was studied by Wanag et al. with the use of TiO_2_ modified by reduced graphene oxide irradiated by artificial solar light [22]. Both studies mentioned showed a significant reduction in bacteria after treatment with TiO_2_-based materials.

Herein, we present the preparation and physicochemical characterization of a nanocomposite material consisting of TiO_2_ and gallic acid (3,4,5-trihydroxybenzoic acid, GA). It is worth noting that GA is a phenolic acid naturally occurring in many fruits and medicinal plants, which can be isolated using various chromatographic methods from different plant species such as *Quercus* spp. and *Punica* spp. However, commercially available gallic acid is produced by the hydrolysis of tannic acid using tannase—a glycoprotein esterase [23]. Gallic acid is commonly applied in the food and pharmaceutical industries. In medical applications, it reveals diverse health-promoting effects as an antioxidant and an anti-inflammatory agent, as well as it also possesses antineoplastic properties. Recently, some reports on its therapeutic activities in gastrointestinal, neuropsychological, metabolic and cardiovascular disorders, as well as its antibacterial activities, have been published [24,25]. Significantly, GA is a strong antioxidant used for in vitro wound healing studies, in which accelerated cell migration (keratinocytes and fibroblasts) was observed [26]. It is important to note that gallic acid-modified nanomaterials, such as gold [27] and magnetite nanoparticles, were studied for their antibacterial activity [28]. To our knowledge, the combination of titanium oxide nanoparticles with gallic acid has not yet been investigated in the context of wound healing. Herein, we present a broad study on gallic acid-modified titania nanoparticles. The novel hybrid material was assessed in terms of its antioxidant, antimicrobial and cytotoxic activities, revealing potential applications as a component for the treatment of hard-to-heal wounds.

## 2. Materials and Methods

### 2.1. Preparation of TiO_2_-GA Nanomaterial

All the reagents and solvents used in this study were purchased from commercial suppliers (Sigma-Aldrich, St. Louis, MI, USA, Fluorochem, Glossop, UK) and were used without additional purification. TiO_2_-GA was prepared by stirring Aeroxide P25 titanium(IV) oxide nanoparticles (200 mg) with 3,4,5-trihydroxybenzoic acid (200 mg) in methanol (40 mL) at room temperature for 24 h without access to light. Next, the suspension was centrifuged (1 h, 5800 rpm, MPW-352 centrifuge). The supernatant was discarded, and the beige precipitate was resuspended in methanol and dried under reduced pressure at ambient temperature. The nanomaterial was stored at room temperature in darkness and with limited access to air.

### 2.2. Physicochemical Characterization of the Material

X-ray diffraction (XRD) analysis was performed using a Pro Philips X’PERT diffractometer (Amsterdam, The Netherlands) with an X’Celerator Scientific detector (CuKα1, wavelength 1.54056 Å, 2Theta angle range 5–90°, scan step size 0.020°). Thermogravimetric analysis (TG) was performed using a Netzsch Jupiter STA 449 F5 Thermoanalyzer with an autosampler coupled with a Bruker Optik FT-IR spectrometer Vertex 70 V (Billerica, MA, USA). ATR-FTIR spectra were recorded with the Perkin Elmer ATR-FTIR Spectrum Two spectrometer (Waltham, MA, USA), using the ATR solids attachment with a diamond crystal. Raman spectra were recorded with the Senterra confocal Raman microscope by Bruker Optik. SEM images were taken using a 1430 VP microscope by LEO Electron Microscopy Ltd. TEM images were taken using a FEI Europe Tecnai F20 X-Twin microscope with atomic resolution. The hydrodynamic diameter of the nanoparticles was measured using the Malvern Panalytical NanoSight LM10 particle size analyzer (Malvern, UK). UV-Vis spectra were recorded on a Jasco V-770 spectrophotometer. An ASAP 2420 apparatus (Norcross, GA, USA) was used to analyze the BET surface area (absorptive: nitrogen, temperature: 77.350 K).

### 2.3. DPPH Antioxidant Assay

The DPPH assay was performed according to protocols available in the literature [28,29]. Briefly, 0.2 mM (200 µM) DPPH solution was prepared by dissolving 7.91 mg DPPH (2,2-diphenyl-1-picrylhydrazyl) in 100 mL of methanol. During the test, 3 mL of DPPH solution (0.2 mM) and 1 mL of the solution containing the appropriate amount of the tested substance were mixed. In this way, the molar concentration of DPPH of approximately 0.05 mM (50 µM) was obtained, which corresponded to the UV-Vis absorbance (517 nm) at approximately 0.6. The preparation of samples was performed under dim light. After 30 min of incubation in the dark, absorbance measurements were taken. All the experiments were performed in duplicate. In the case of TiO_2_ and TiO_2_-GA, sonication (10 s in Chemland ultrasonic cleaner, 180 W) was used to homogenize the sample to maintain the reproducibility of the results. In the case of TiO_2_ and TiO_2_-GA, the materials were separated from the solution using syringe filtration (PureLand 0.22 µm Nylon Syringe Filters, Chemland, Stargard, Poland) right before UV-Vis measurements. The radical scavenging activity was calculated using the equation below:

(1) $$\begin{array}{c} I\;\left(\%\right)=\frac{\left(\mathrm{Ac}-\mathrm{As}\right)}{\mathrm{Ac}}\times 100\; \end{array}$$

I (%)—the percentage of inhibition.
As—the absorbance of the compounds.
Ac—the absorbance of the DPPH solution (control).

To determine the IC_50_ value (concentration required to achieve 50% inhibition of the DPPH radical) of the materials, the I (%) was plotted against different concentrations of the materials (TiO_2_-GA, TiO_2_) or reference compounds (GA, EGCG, curcumin, ascorbic acid). The IC_50_ was calculated from the linear equation obtained with the least-squares method.

### 2.4. Microtox Assay

An acute toxicity test was performed on a Modern Water Microtox Model 500 (Modern Water, Cambridge, UK) equipped with Modern Water MicrotoxOmni 4.2 software (Modern Water, Cambridge, UK) following the procedure provided by the supplier. The change in the bioluminescence of the bacterial suspension was monitored upon the addition of the sample suspension. A decrease in cell viability was calculated based on the decrease in bioluminescence detected in comparison to the negative control. For all the experiments, the material suspensions of appropriate concentrations were prepared using deionized water, followed by sonication and vortexing to obtain uniform dispersions at the time of testing.

### 2.5. Microbial Strains and Cultivation

Microbial strains of *Staphylococcus aureus* ATCC 25923, *Escherichia coli* ATCC 25922 and *Candida albicans* ATCC 10231 were used. All strains were stored in Microbank cryogenic vials at −70 ± 10 °C. Before each experiment, bacterial and fungal subcultures were prepared on Tryptone Soya Agar (TSA; OXOID, Basingstoke, UK) or Sabouraud Dextrose Agar (SDA; OXOID, UK), respectively. They were grown at 36 ± 1 °C for 18–24 h, and then a single colony from the plate was inoculated in Brain–Heart Infusion Broth (BHI; OXOID, UK) or Sabouraud Dextrose Liquid Medium (SDLM; OXOID, UK), respectively, and incubated aerobically at 36 ± 1 °C for 18–24 h. Next, microbial cells were centrifuged (3000 rpm for 15 min at 4 °C), resuspended in 0.9% NaCl (pH = 7.0) and diluted with 0.9% NaCl to a final concentration of ca. 10^7^ colony-forming units (CFU) per mL. This suspension was further used in the experiments.

N values of microbial cells in CFU/mL were calculated according to the formula below. Afterward, to improve the clarity of the data, the N values were logarithmized.

(2) $$\begin{array}{c} N=\left(\frac{\sum \;c}{V\times \left(n_{1}+0.1\times n_{2}\right)\times d_{1}}\right)\; \end{array}$$

N—number of CFU/mL.
∑ c—sum of colonies on counted plates.
V—inoculated volume [mL].
n_1_—number of plates from the first calculated dilution.
n_2_—number of plates from the second calculated dilution.
d_1_—dilution factor corresponding to the first dilution taken into account.

### 2.6. Photocytotoxicity Assessment against Bacteria and Fungi

Antimicrobial activity parameters (MIC and MBC) of the tested materials against *S. aureus* ATCC 25923, *E. coli* ATCC 25922 and *C. albicans* ATCC 10231 were initially determined using the broth microdilution method [30]. In the next experiment, the same three microorganisms were involved in order to provide basic information about the light-induced antimicrobial activity of the tested materials against different types of microorganisms such as Gram-positive bacteria, Gram-negative bacteria and fungus, respectively. To each well of the microtiter plate was added: (a) an aliquot of 150 μL of microbial suspension in sodium chloride solution (isotonic 0.9% NaCl) containing approximately 10^7^ CFU/mL and (b) 150 µL of a suspension containing photosensitizer (TiO_2_ or TiO_2_-GA) at the concentration of 2 mg/mL (Table 1). The control sample contained 150 µL of 0.9% NaCl instead of the photosensitizer suspension. The suspension of the microbes with the photosensitizer was incubated for 20 min at room temperature. After that, the microtiter plates with samples were irradiated for 2 h with homemade LED lamps emitting light of either λ_max_ = 365 nm or λ_max_ = 425 nm from the distance of 1 cm. After this time, volumes of 1 µL and 50 µL from the undiluted samples were seeded on an appropriate solid growth medium (Tryptone Soya Agar, Sabouraud Dextrose Agar, Oxoid Ltd., Basingstoke, UK). After incubation (36 ± 1 °C for 24 h), the number of colonies was counted, and the number of bacteria surviving the treatment was determined. The results were expressed as log CFU/mL.

In order to determine the optimal conditions in the photocytotoxicity study, the experiment with *S. aureus* was carried out. Approximately 10^7^ CFU/mL of microorganisms suspended in 150 µL of 0.9% NaCl and 150 µL of photosensitizer (TiO_2_-GA) suspension (2 mg/mL) were added to the wells of the microtiter plate (Table 2). The negative control sample contained 150 µL of 0.9% NaCl instead of the photosensitizer suspension. Preincubation time was set to 20 min. Microtiter plates with samples were irradiated from 10 to 120 min with 425 nm light from 1 cm. The light dose was measured with RD 0.2/2 radiometer (Optel) at 70 mW/cm^2^. After the mentioned time, the samples were 1:10 serially diluted, and 50 µL from the undiluted sample and 100 µL from each dilution were seeded on an appropriate solid growth medium (Tryptone Soya Agar, Oxoid Ltd. UK). Plates were incubated at 36 ± 1 °C for 24 h. After this time, the visible colonies were counted, and for convenience, the results were expressed as log CFU/mL. The experiment was conducted with and without access to light. The experiment was performed in duplicate.

### 2.7. Cell Culture

MRC-5 pd19 cells were cultured under standard conditions, at 37 °C, in an atmosphere enriched with 5% CO_2_, saturated with a water vapor incubator (Binder, Tuttlingen, Germany). The basic culture medium was Dulbecco’s Modified Eagle’s Medium (DMEM) (Biowest, Nauille, France) supplemented with 10% fetal bovine serum (FBS) (Biowest, Nauille, France) with the addition of 2 mM L-glutamine and 5% non-essential amino acid solution. Cell culture, which reached 80–90% confluence, was passaged every 3–4 days. All experiments were carried out in sterile conditions under a biosafety cabinet with laminar airflow (Telstar, Madrid, Spain).

### 2.8. MTT Assay

MRC-5 pd19 cells were seeded onto 96-well flat-bottomed plates at a concentration of 10,000 cells/well. TiO_2_, TiO_2_-GA or GA was suspended/dissolved in the completed culture medium at the final concentrations of 0.01%, 0.10% and 1.00% at the final volumes of 200 µL per well. After 24 h, each of the reagents was added to the cells. Control cells were not treated with synthesized reagents; they were incubated with completed cell culture medium. The incubation of cells with TiO_2_, TiO_2_-GA or GA lasted 24 or 48 h. Then, the medium was discarded, and the new medium containing MTT (3-(4,5-dimethylthiazol-2-yl)-2,5-diphenyltetrazolium bromide) (Affymetrix, Cleveland, OH, USA) at a final concentration of 0.5 mg/mL was added to the cell culture. Cells were incubated for 2.5 h in cell culture conditions. Next, the medium was removed, and 100 µL of DMSO (Thermo Scientific, Waltham, MA, USA) was added to dissolve the formed formazan crystals. The absorbance was read with a Multiskan plate reader at 570 nm, background 655 nm (Thermo Scientific, Waltham, MA, USA). The experiment was performed in triplicate.

## 3. Results and Discussions

### 3.1. Synthesis and Characterization of TiO_2_-GA Nanomaterial

The TiO_2_-GA nanomaterial was prepared using the chemical deposition method with the excess of gallic acid on TiO_2_ [11,28,31,32,33]. Gallic acid contains hydroxyl and carboxyl groups that can coordinate with titania’s surface hydroxyl groups.

#### 3.1.1. Surface Morphology

**Figure 1:**
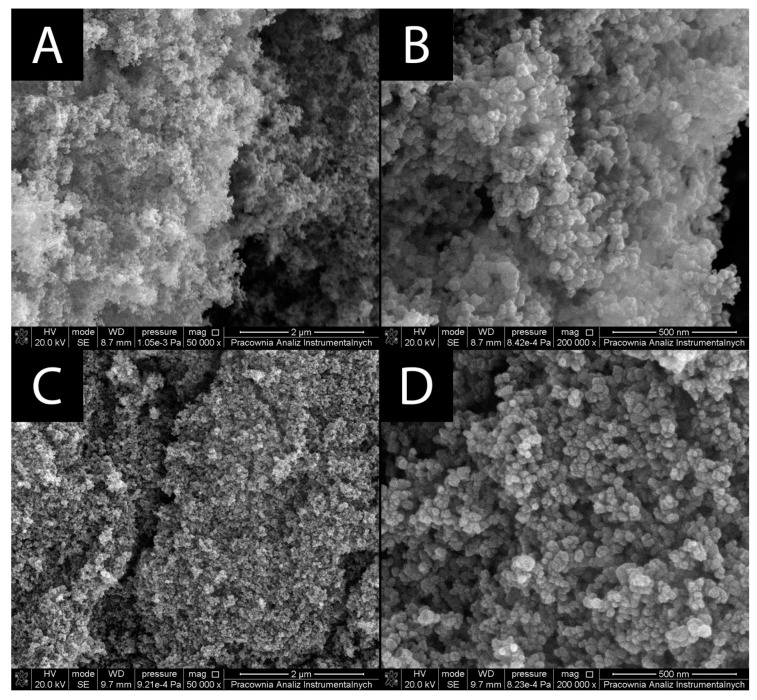
SEM images of prepared nanomaterials. (**A**,**B**) TiO_2_, (**C**,**D**) TiO_2_-GA.

The surface morphology of the prepared nanomaterials was characterized by SEM (Figure 1). A very characteristic feature is a high tendency for aggregation of TiO_2_ and TiO_2_-GA. In both cases, the aggregates appear to be over 200 nm in size. The shape of the particles appears to be semispherical.

**Figure 2:**
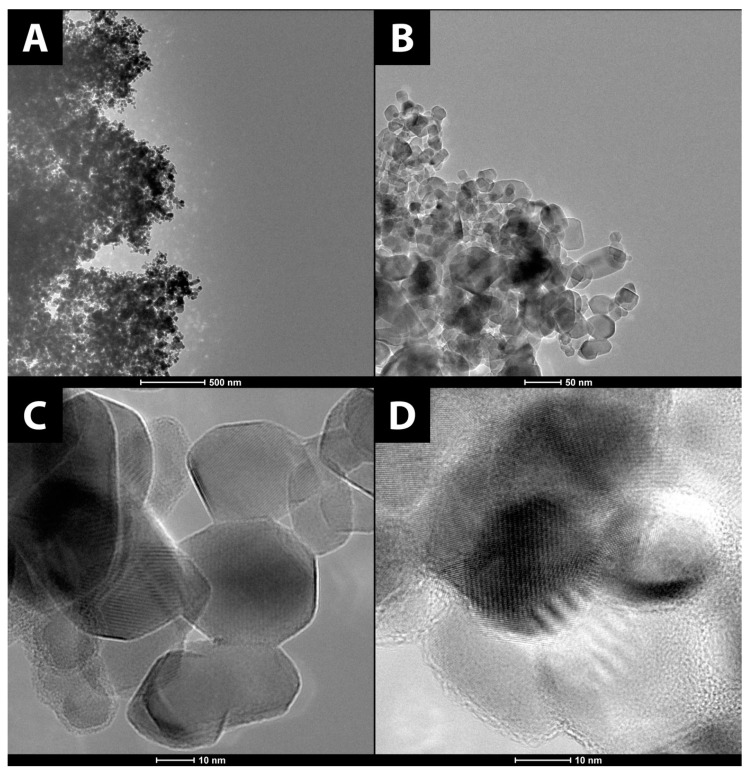

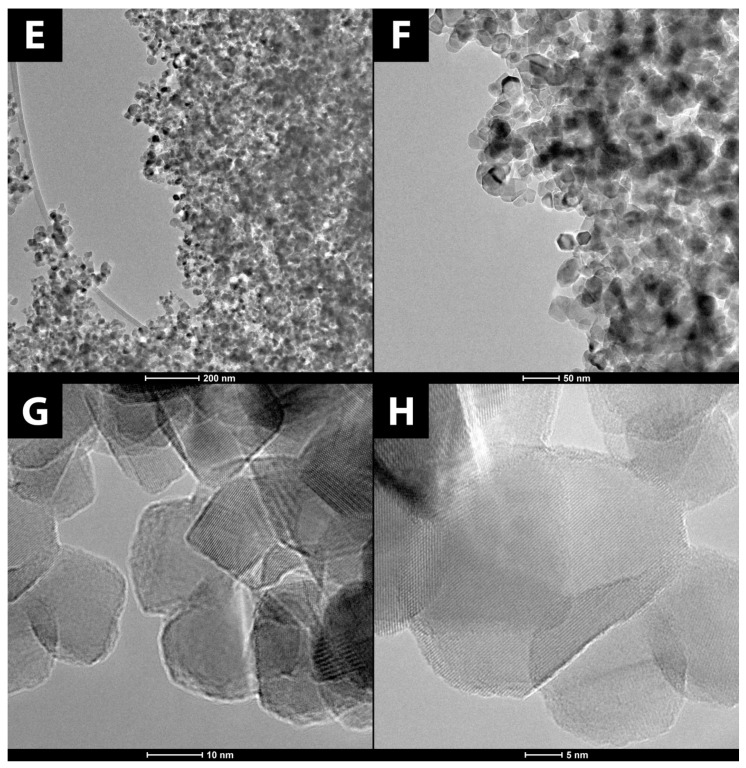
TEM images of (**A**–**D**) TiO_2_ and (**E**–**H**) TiO_2_-GA.

In addition to SEM, HR-TEM images were also taken (see Figure 2), confirming the previous observations regarding aggregation [34]. HR-TEM allowed for capturing images with much higher resolutions and magnifications than SEM. The micrographs reveal slight deviations from the declared grain size of 20 nm for some of the particles, possibly as a consequence of shape irregularities. The particles were found to be monodisperse. Anatase particles appear to be semispherical in shape, whereas rutile is hexagonal with some irregularities. Images taken with higher magnification reveal lattice fringes characteristic for polycrystalline material such as anatase and rutile, which were used in this study. Individual grain sizes were measured, revealing that the particles are around 20 nm in size, which falls in line with the size declared by the manufacturer (21 nm). The material was also characterized using nanoparticle tracking analysis (NTA).

**Figure 3:**
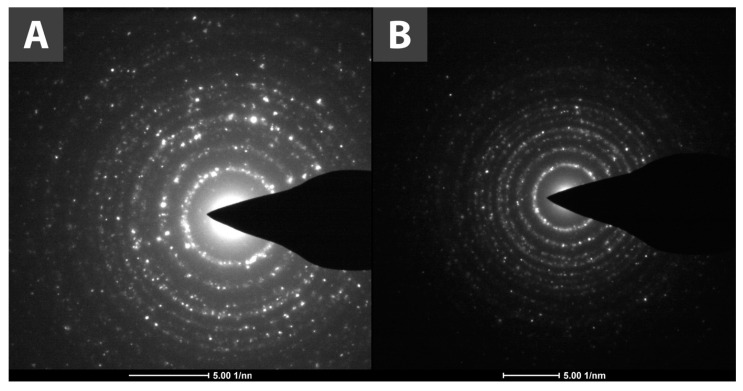
SAED patterns of (**A**) TiO_2_ and (**B**) TiO_2_-GA.

Further application of the HR-TEM technique allowed for capturing selected-area diffraction (SAED) patterns shown in Figure 3. Reflections specific to anatase and rutile [35] are presented on the diffractograms and correspond with the reflections observed in the X-ray powder diffraction (XRPD) measurements.

#### 3.1.2. Nanoparticle Tracking Analysis

Both materials, bare TiO_2_ and TiO_2_-GA, were analyzed using the NanoSight^®^ LM10 apparatus, which allowed for determination of the hydrodynamic diameter of the particles (Table 3). The measurements were conducted in distilled water and reflected actual particle sizes in an aqueous environment. The mean size of TiO_2_ nanoparticles equals 255.7 nm with a standard deviation (SD) of 74.5 nm. Functionalization of TiO_2_ with gallic acid seems to reduce the tendency to form aggregates, as the mean size for TiO_2_-GA nanoparticles was notably lower (218.0 nm, SD 74.9 nm). Polydispersity indices (PDI) calculated for both materials were lower than 0.2, which confirms their monodisperse nature [36].

#### 3.1.3. XRPD Analysis

**Figure 4:**
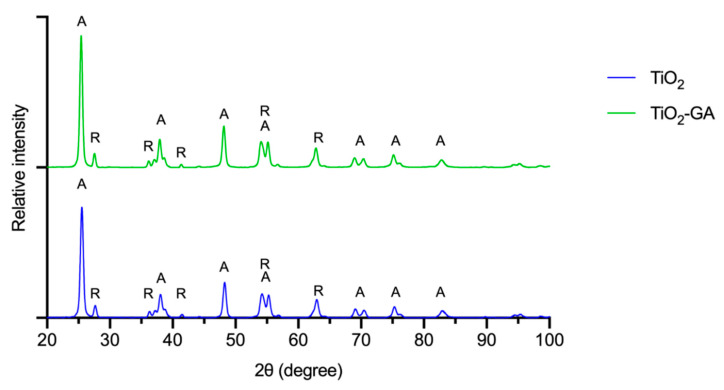
XRPD patterns of TiO_2_ and TiO_2_-GA. Labels describe peaks belonging to anatase (A) and rutile (R).

The crystallinity of the materials was analyzed with XRPD (Figure 4). The obtained XRPD patterns show reflections characteristic for anatase (JCPDS card no. 21-1272) at 25.53, 38.06, 48.27, 54.22, 55.29, 69.11, 70.48, 75.31 and 82.93°, with additional reflections at 27.67, 36.31, 41.47, 54.22 and 62.94° coming from the admixture of rutile (JCPDS card no. 21-1276) present in bare TiO_2_ [37]. No reflections for GA were observed. As such, it can be concluded that the functionalization of titania with GA had no impact on the crystalline structure of TiO_2_ within the hybrid material.

#### 3.1.4. Infrared Spectroscopy

**Figure 5:**
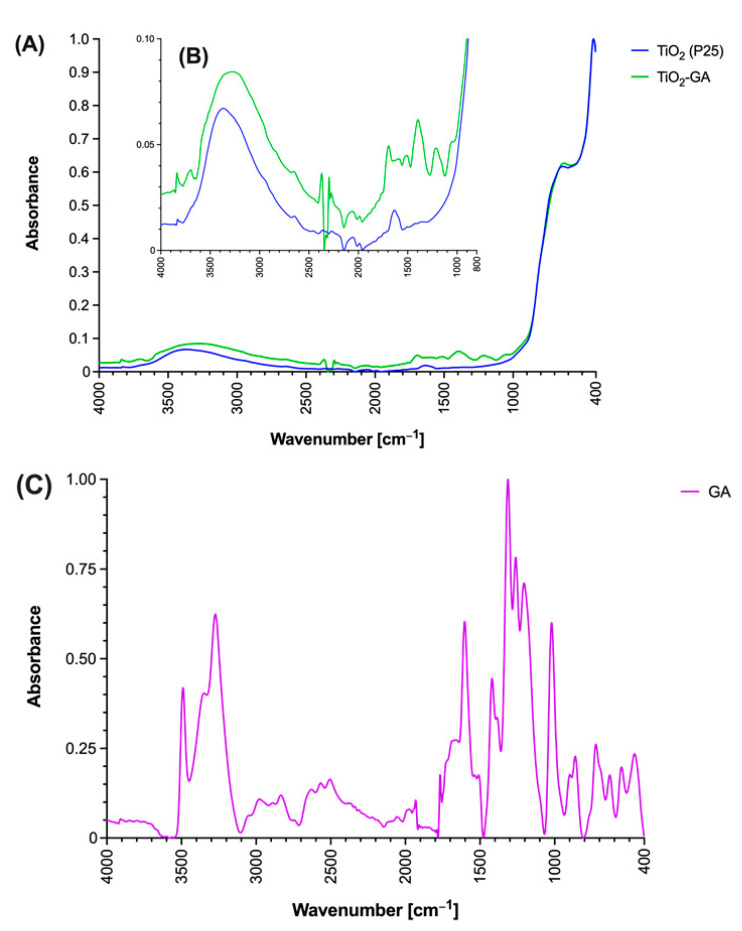
ATR-FTIR spectra TiO_2_, TiO_2_-GA and gallic acid. (**A**) Full spectrum in the range of 4000–400 cm^−1^, (**B**) inset of the 4000–800 cm^−1^ range with relative absorbance below 0.1. (**C**) Spectrum of gallic acid in the range of 4000–400 cm^−1^.

In the ATR-FTIR spectrum of GA, characteristic bands for O-H and C=O groups were noted (3500 and 1800–1600 cm^−1^). Bare TiO_2_ and TiO_2_-GA were also subjected to ATR-FTIR spectroscopy measurements (Figure 5). Both materials revealed bands specific to Ti-O bond vibrational mode at 643.74 cm^−1^ and a band at 416.99 cm^−1^ from Ti-O-Ti bridging stretching mode [38]. At 1630 cm^−1^, a band coming from O-H bending vibrations of chemisorbed water was noted. The broadband in the range between 3500–3000 cm^−1^ comes from O-H stretching vibrations, and this band is more pronounced in TiO_2_-GA due to the abundance of hydroxyl groups within the hybrid material. Additionally, in the spectrum of TiO_2_-GA, two new bands at 3836.14 and 3699.18 cm^−1^ were noted, which are not present in TiO_2_, and were assigned to hydroxyl groups O-H stretching vibrations of GA. For TiO_2_-GA, the bands noted within the 1600–1400 cm^−1^ range belong to the aromatic ring of GA, whereas the bands appearing within the 1800–1600 cm^−1^ and 1400–1000 cm^−1^ ranges result from the hydroxyl and carboxyl groups’ presence.

#### 3.1.5. Raman Spectroscopy

**Figure 6:**
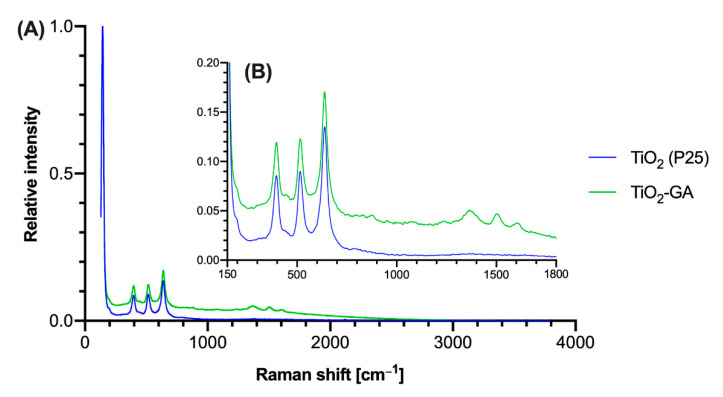
Raman spectra of analyzed materials TiO_2_ and TiO_2_-GA. (**A**) Full spectrum in the range of 4000–0 cm^−1^, (**B**) presents inset in the range of 1800–150 cm^−1^ with relative intensity below 0.2.

Both materials, bare TiO_2_ and TiO_2_-GA, were analyzed with Raman spectroscopy (Figure 6). An analysis of the spectra revealed the presence of signals characteristic for anatase and rutile TiO_2_ at the following wavelengths: 144.0, 396.18, 516.56 and 638.07 cm^−1^ [39]. In the case of the TiO_2_-GA spectrum, additional bands specific for GA were also noted at 1364.9, 1502.1 and 1609 cm^−1^ [40]. The TiO_2_-GA spectrum showed a notably higher baseline, which could result from the sample heating up.

#### 3.1.6. Thermogravimetric Analysis

Thermal analysis of TiO_2_ (Table 4) revealed 1.04% mass change within the range of 29–222 °C. The first stage was associated with the evaporation of surface water, as well as condensation and evaporation of adsorbed hydroxyl groups [41]. Interestingly, the second stage, which began at 389 °C and continued up to the end temperature of 800 °C, showed 0.89% mass gain. As the analysis was performed in the air atmosphere, this effect was associated with oxidation reactions occurring at the material surface at higher temperatures. This effect was observed for bare TiO_2_ and TiO_2_-GA.

The first stage of GA thermal analysis started at 73 °C and lasted until 100 °C, which is associated with the evaporation of adsorbed water molecules, resulting in a mass loss of 3.02%. The second stage fell into the range of 248–301 °C, where thermal degradation of GA itself begins and, in this stage, 61.45% of the mass was lost. The third stage occurred in the range of 301–357 °C, resulting in a mass deficit of 12.75%. The last, fourth, stage occurred in the range of 357–597 °C and resulted in 22.23% mass loss. GA was entirely degraded within the tested temperature range.

Similar to TiO_2_, for TiO_2_-GA, the first stage was attributed to the desorption of surface water and hydroxyl groups within the range of 29–205 °C and resulted in 0.92% mass loss. The next stage was associated with the degradation of GA and appeared in the range of 205–398 °C, resulting in a total 2.15% mass deficit in this stage. The third stage was observed from 457 °C to 800 °C, where 0.51% mass gain was noted.

### 3.2. Antioxidant Activity

**Figure 7:**
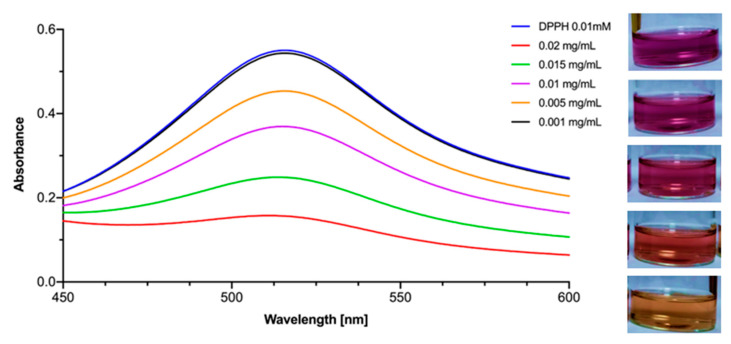
Absorbance of DPPH during DPPH assay of TiO_2_-GA depending on the concentration of the latter and photographs of the DPPH solutions with the addition of different concentrations of TiO_2_-GA suspensions.

The antioxidant properties of the tested nanomaterial were determined using the DPPH assay [29]. During this assay, a change in the color of the methanolic DPPH (2,2-diphenyl-1-picrylhydrazyl) solution from deep violet to pale yellow is observed in the presence of an antioxidant. This occurs due to the action of the antioxidant agent, which causes the transformation of the stable DPPH violet radical into a pale yellow non-radical derivative (see Figure 7). To evaluate the antioxidant properties of TiO_2_, TiO_2_-GA and GA, serial dilutions of the materials were prepared, and the IC_50_ value was calculated. To put the obtained data in context, three common antioxidants were also tested: ascorbic acid (vitamin C), (–)-epigallocatechin 3-*O*-gallate (EGCG) and curcumin. The summarized results of the DPPH test are presented in Table 5. On the one hand, it was found that pristine TiO_2_ nanoparticles do not exert any antioxidant activity. On the other hand, GA is a strong antioxidant (IC_50_ equals 0.910 ± 0.002 mg/L). By combining both materials in TiO_2_-GA, the resulting composite retains most of the antioxidant properties of GA with IC_50_ at the level of 13.5 ± 0.3 mg/L. It is worth noting that the antioxidant properties of the nanomaterial do not differ a lot from those of reference antioxidants such as vitamin C, EGCG or curcumin.

### 3.3. Antimicrobial Activity

#### 3.3.1. Microtox Acute Toxicity Evaluation

The Microtox acute toxicity test is routinely used to determine the ecotoxicity of solutions. In the test, the effect is related to the bioluminescence of the *Aliivibrio fischeri* bacteria, which emit light at 490 nm. The decrease in the bioluminescence caused by any substance or solution reflects the metabolism, and thus the cell viability [42]. However, as a biological factor used in this test, *A. fischeri* represents a Gram-negative bacteria, which could be considered in the preliminary evaluation of the antimicrobial properties of tested materials, compounds or plant extracts [43,44,45]. Due to the specificity of the test, the results may be biased, as the cell walls of the used bacteria are broken for more rapid screening. Therefore, the internalization of the compounds into the bacterial cells is omitted as compared to normal bacterial cells. Thus, the obtained results may not fully reflect the antibacterial activity, as the effect exerted on the intracellular targets is independent of the particle size.

**Figure 8:**
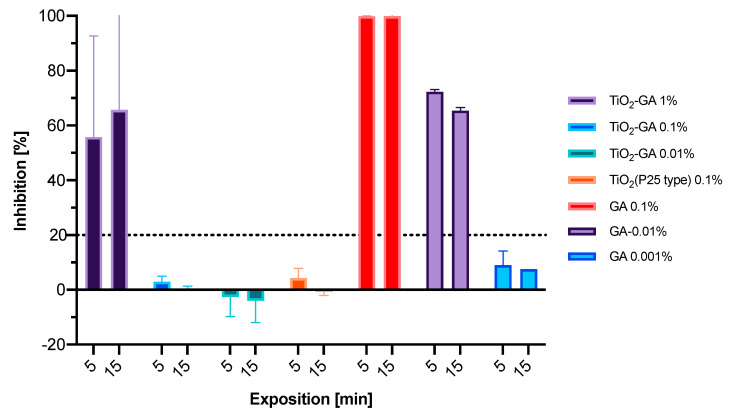
*A. fischeri* bioluminescence inhibition upon incubation with solutions/suspensions of the tested materials (mean values ± standard deviation). The dashed line represents the 20% threshold, the arbitrary value of compound toxicity. All experiments were performed in triplicate.

The suspensions of TiO_2_-GA and TiO_2_ and solutions of GA were subjected to Microtox evaluation, the results of which are summarized in Figure 8. It was found that the functionalized titania exerted the same effect as pristine TiO_2_ nanoparticles. This was probably the result of the immobilization of GA on the surface of TiO_2_, which did not allow GA for easy interaction with the cellular components of the bacteria. Gallic acid alone was highly active, even in much lower concentrations. The “toxic” concentration (20% cell viability decrease, EC_20_) was calculated at 0.3347% for TiO_2_-GA and 0.0026% for GA alone, which indicates that the functionalized material is almost 131 times less active after 5 min exposure.

#### 3.3.2. Photocytotoxicity against Bacteria and Fungi

The first experiment was performed to determine the antimicrobial activity (MIC and MBC values) of materials against Gram-positive bacteria *S. aureus,* Gram-negative bacteria *E. coli* and fungus *C. albicans*. According to the obtained results, neither TiO_2_ nor TiO_2_-GA revealed significant antimicrobial activity in the tested range of concentrations (data not shown). In the second experiment, the photocytoxicity of materials on the above-indicated microorganisms was assessed in two different irradiation conditions at 365 nm and 425 nm (Table 6). In this experiment, no phototoxicity was noted towards *C. albicans*. In the case of *E. coli* and *S. aureus,* a reduction in viable cells was observed. The maximum reduction in *E. coli* growth was achieved using TiO_2_-GA and lamp irradiation at 425 nm, which allowed for reaching approx. 4.1 log CFU/mL. At the same time, it was observed that light alone reduced the number of viable bacteria by about 1.2 log CFU/mL. At 425 nm, the sample containing TiO_2_ showed much less activity, equal to about 2.1 log CFU/mL. Interestingly, the reduction in survival of the *E. coli* strain after exposure to 365 nm light generated the opposite results. Namely, the sample with bare TiO_2_ reduced the number of viable bacteria by about 1.3 log CFU/mL, and the sample with TiO_2_-GA did not reveal any effect.

In the case of *S. aureus*, the results turned out to be more expressive. The reduction in the viable bacteria exposed to TiO_2_-GA and light at 425 nm was approximately 4.2 log CFU/mL. The effect of TiO_2_ alone was not significant after exposition to the light of this length. At the wavelength of 365 nm, the observed reduction in *S. aureus* survival after incubation with TiO_2_ was about 3.1 log CFU/mL, whereas TiO_2_-GA did not show any effect.

As the best effect was observed in the case of *S. aureus*, the following experiment aiming to determine the time necessary for the above-described phototoxic effect to take place was designed. The selected experimental parameters were: *S. aureus*, TiO_2_-GA 1 mg/mL, 425 nm light.

**Figure 9:**
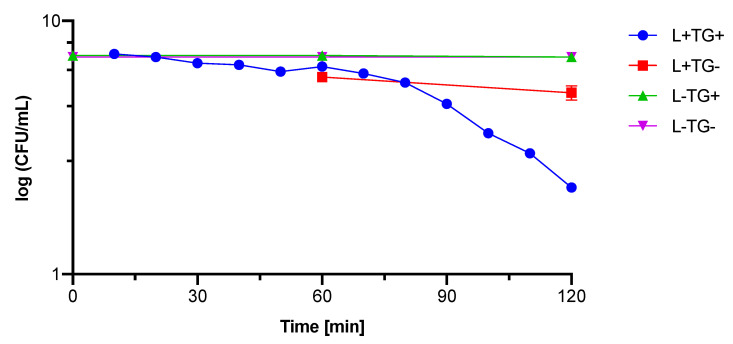
The inactivation of *S. aureus* with TiO_2_-GA and visible light (λ = 425 nm) irradiation in function of time. Where: L+ is the irradiated sample; L− is the non-irradiated sample (tested in the dark); TG+ is the sample with photosensitizer (TiO_2_-GA); TG−is the sample without photosensitizer.

In the third experiment, the survival of *S. aureus* was assessed in presence of TiO_2_-GA (1 mg/mL) at 425 nm light (Figure 9). The exposure of *S. aureus* to those conditions resulted in the reduction of its survival by 5 log CFU/mL after 120 min of irradiation. The light alone reduced the viability of bacteria by 2 log CFU/mL after 120 min of irradiation. No dark toxicity—the toxicity of the nanomaterial itself, without irradiation—was observed during the experiment. The synergism of the combined action of light and nanomaterial started after 80 min of irradiation. The added value of irradiation with the simultaneous presence of the photosensitizer was estimated at 3 log CFU/mL.

### 3.4. Toxicity Assessment

**Figure 10:**
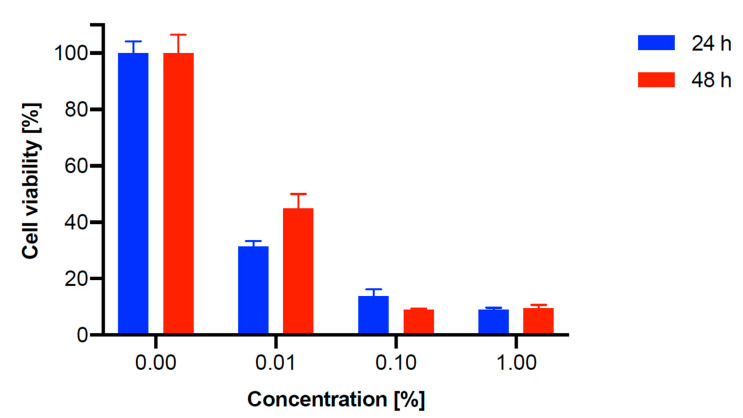
MTT assay on MRC-5 pd 19 cell line (human fibroblasts) after 24 and 48 h incubation for TiO_2_-GA nanomaterial.

The MTT viability test was performed to investigate the toxicity effect of TiO_2_-GA on human fibroblast cells (MRC-5 pd 19). The metabolic activity of MRC-5 pd 19 was analyzed as a percentage of cell viability, and TiO_2_-GA concentrations in the range of 0.01–1% at two time points, 24 and 48 h, were used. In the studied concentrations, gallic acid revealed significant cytotoxicity against human fibroblasts with IC_50_ values below 0.01% after 24 h of incubation. The level of metabolic activity decreased with the increasing concentration of TiO_2_-GA, which is presented in Figure 10. It is worth noting that the cytotoxicities of TiO_2_ and TiO_2_-GA are comparable.

Although titanium(IV) oxide is considered a safe food additive (E171) and is used in numerous preparations for medical and cosmetic applications, it is also necessary to decide whether its nano form is as safe for medical applications [18,46]. Potential TiO_2_ toxicity is known to induce the production of reactive oxygen species (ROS), thus resulting in oxidative stress through the cells [47] or apoptosis [48]. TiO_2_ has been widely studied on various cell lines, such as rat and human glial cells [48] or human osteoblast cell-like cells [49]. Some research groups [50] have analyzed the biocompatibility of TiO_2_ using its concentrations, such as those found in physiological body fluids, and investigated the effect of cell toxicity. It is difficult to form a straightforward conclusion about TiO_2_ biological effects because tested models differ greatly, and the concentrations of TiO_2_ were differentiated. Apart from biological issues, technical ones should be considered, as TiO_2_ is a hydrophobic substance. Therefore, it is challenging to study the reagent, which is insoluble in culture media.

As the herein presented hybrid material consists of TiO_2_ and GA, some methods need to be optimized in future experiments. A promising direction for further research will be such functionalization of TiO_2_, which will increase the stability of the formed suspensions and reduce the aggregation of nanoparticles. Defining the “biocompatibility index” [51] to assess the suitability of TiO_2_-GA as an antiseptic agent was also planned. Unfortunately, fibroblast cells were detaching from the wall of the bottle after 40 min of irradiation in conditions corresponding to those used for bacteria (2 h of irradiation with a 425 nm lamp), which made taking measurements impossible. The described effect on fibroblasts is most likely caused by oxidative stress [52,53]. In future studies, it would be advantageous to modify the material further to strengthen the antibacterial photocytotoxic effect after irradiation with visible light. This can probably be achieved by using other compounds for surface functionalization or thanks to doping of the titanium oxide itself. Irradiation with blue light, despite its antibacterial action, should be avoided due to its unfavorable influence on fibroblasts.

## 4. Conclusions

In the herein presented study, a nanomaterial based on titanium oxide functionalized with gallic acid was obtained and characterized. The level of gallic acid absorption on the surface of titanium oxide was estimated at 2.5% based on thermogravimetry results. The Microtox assay revealed a lack of ecotoxicity of TiO_2_-GA at concentrations up to 0.1%. The DPPH assay showed a significant increase in the antioxidant properties of TiO_2_-GA (IC_50_ = 13.5 ± 0.3 mg/L) compared to titanium(IV) oxide, which had no antioxidant effect. Interestingly, the value assigned to the nanomaterial corresponds to other popular antioxidants such as curcumin or vitamin C (IC_50_~2–4 mg/L). The phototoxic effect of the nanomaterial against *S. aureus*, *E. coli* and *C. albicans* was thoroughly studied, with the most significant reduction in bacterial survival by 5 log CFU/mL noted for *S. aureus* irradiated with 425 nm light (~70 mW/cm^2^). The MTT test was performed to determine the toxicity of the nanomaterial to human fibroblast cells. In the tested concentrations (0.01–1%), gallic acid presented significant cytotoxicity against human fibroblasts, with IC_50_ values below 0.01% after 24 h of incubation, whereas the cytotoxicities of TiO_2_ and TiO_2_-GA were comparable.

TiO_2_-GA presented a bimodal effect in this way, that in the presence of blue light, it revealed antibacterial properties, whereas in the dark, it demonstrated antioxidant properties. This prompted us to conclude that TiO_2_-GA could be an additive to dressings and rinsing suspensions for treating difficult-to-heal wounds. Gallic acid as a tanning agent has an astringent effect—it binds to skin proteins, causing their inactivation. The low content of GA in the TiO_2_-GA nanomaterial may result in the normalization of the healing processes by slowing it down and remodeling existing but damaged fibers. Moreover, there are reports that TiO_2_ can improve wound healing by adsorbing proteins on the surface and reducing inflammation. As a result, the material can reduce the formation of keloids and improve the wound appearance after fusion [54,55]. To sum up, the research carried out on TiO_2_-GA indicates that it could be considered a new and valuable additive in the treatment of difficult-to-heal wounds, but further research is necessary to fully understand the mechanisms and phenomena behind the presented activity.

## Figures and Tables

**Table 1:** Amounts of ingredients added to the wells of a microtiter plate during an experiment involving 3 strains of microorganisms.

	L− T+	L− TG+	L− PS−	L+ T+	L+ TG+	L+ PS−
10^7^ CFU/mL microorganisms	150 µL	150 µL	150 µL	150 µL	150 µL	150 µL
0.9% NaCl			150 µL			150 µL
2 mg/mL TiO_2_-GA (TG)		150 µL			150 µL	
2 mg/mL TiO_2_ (T)	150 µL			150 µL		

Where: L+ is the irradiated sample; L− is the non-irradiated sample (tested in the dark); PS−is the sample without photosensitizer (neither TiO_2_-GA nor TiO_2_).

**Table 2:** Amounts of ingredients added to the wells of a microtiter plate during the experiment on *S. aureus*.

	L− TG+	L− PS–	L+ TG+	L+ PS−
Time points, min	0, 60, 120	0, 60, 120	10, 20, 30, 40, 50, 60, 70, 80, 90, 100, 110, 120	60, 120
10^7^ CFU/mL microorganisms	150 µL	150 µL	150 µL	150 µL
0.9% NaCl		150 µL		150 µL
2 mg/mL TiO_2_-GA (TG)	150 µL		150 µL	

Where: L+ is the irradiated sample; L− is the non-irradiated sample (tested in the dark); PS− is the sample without photosensitizer.

**Table 3:** Nanoparticle size measurements with NTA and BET surface area.

Name	Mean [nm]	SD	PDI	S_BET_ [m^2^/g]
TiO_2_	255.7	74.5	0.085	57
TiO_2_-GA	218.0	74.9	0.118	61

PDI was calculated according to formula (SD/Mean)^2^.

**Table 4:** Results of thermogravimetric analysis of TiO_2_ and TiO_2_-GA.

	First Stage			Second Stage			Third Stage			Fourth Stage			Residue
	T_p_ (°C)	T_k_ (°C)	Δm (%)	T_p_ (°C)	T_k_ (°C)	Δm (%)	T_p_ (°C)	T_k_ (°C)	Δm (%)	T_p_ (°C)	T_k_ (°C)	Δm (%)	(%)
TiO_2_	29	222	1.04	389	800	−0.89							99.85
GA	73	100	3.02	248	301	61.45	301	357	12.75	357	597	22.23	0.55
TiO_2_-GA	29	205	0.92	205	398	2.15	457	800	−0.51				97.44

T_p_—onset temperature, T_k_—end temperature, ∆m—change of mass of the analyzed sample.

**Table 5:** IC_50_ values of the tested samples determined in the DPPH test.

Sample (Compound/Material)	IC_50_ ± S.E.M. (mg/L)
TiO_2_	>1000
TiO_2_-GA	13.5 ± 0.3
GA	0.910 ± 0.002
Vitamin C	2.12 ± 0.03
EGCG	1.027 ± 0.003
Curcumin	4.43 ± 0.02

IC_50_—concentration required to achieve 50% inhibition of the DPPH radical; S.E.M.—standard error of the mean.

**Table 6:** Reduction in amount of viable microorganisms in log CFU/mL.

Light	365 nm		425 nm	
Nanomaterial	TiO_2_	TiO_2_-GA	TiO_2_	TiO_2_-GA
*C. albicans*	no effect			
*E. coli*	1.3 log	no effect	2.1 log	4.1 log
*S. aureus*	3.1 log	no effect	1.4 log	4.2 log

## Data Availability

Not applicable.
